# Identification of Novel *Coxiella burnetii* Genotypes from Ethiopian Ticks

**DOI:** 10.1371/journal.pone.0113213

**Published:** 2014-11-25

**Authors:** Kinga M. Sulyok, Sándor Hornok, Getachew Abichu, Károly Erdélyi, Miklós Gyuranecz

**Affiliations:** 1 Institute for Veterinary Medical Research, Centre for Agricultural Research, Hungarian Academy of Sciences, Budapest, Hungary; 2 Faculty of Veterinary Science, Szent István University, Budapest, Hungary; 3 Department of Parasitology, National Research Center, Sebeta, Ethiopia; 4 Veterinary Diagnostic Directorate, National Food Chain Safety Office, Budapest, Hungary; University of Arkansas for Medical Sciences, United States of America

## Abstract

**Background:**

*Coxiella burnetii*, the etiologic agent of Q fever, is a highly infectious zoonotic bacterium. Genetic information about the strains of this worldwide distributed agent circulating on the African continent is limited. The aim of the present study was the genetic characterization of *C. burnetii* DNA samples detected in ticks collected from Ethiopian cattle and their comparison with other genotypes found previously in other parts of the world.

**Methodology/Principal Findings:**

A total of 296 tick samples were screened by real-time PCR targeting the IS*1111* region of *C. burnetii* genome and from the 32 positive samples, 8 cases with sufficient *C. burnetii* DNA load (*Amblyomma cohaerens*, n = 6; *A. variegatum*, n = 2) were characterized by multispacer sequence typing (MST) and multiple-locus variable-number tandem repeat analysis (MLVA). One novel sequence type (ST), the proposed ST52, was identified by MST. The MLVA-6 discriminated the proposed ST52 into two newly identified MLVA genotypes: type 24 or AH was detected in both *Amblyomma* species while type 26 or AI was found only in *A. cohaerens*.

**Conclusions/Significance:**

Both the MST and MLVA genotypes of the present work are closely related to previously described genotypes found primarily in cattle samples from different parts of the globe. This finding is congruent with the source hosts of the analyzed Ethiopian ticks, as these were also collected from cattle. The present study provides genotype information of *C. burnetii* from this seldom studied East-African region as well as further evidence for the presumed host-specific adaptation of this agent.

## Introduction

*Coxiella burnetii*, the causative agent of Q fever, is a zoonotic Gram-negative bacterium with a worldwide distribution. Q fever is typically an acute febrile illness with nonspecific clinical signs in humans, but hepatitis and atypical pneumonia are seen in severe cases, and a small proportion of infected people develop chronic infection with life-threatening valvular endocarditis [1], [2]. Domestic ruminants are considered the primary hosts of *C. burnetii*, but it has been described in a wide range of other animal species including mammals, birds and ticks [3]. Domestic ruminants are often asymptomatic carriers of the bacterium, but the agent may cause abortion in these animals [1]. *C. burnetii* has been detected in about 40 different tick species which may act as reservoirs in nature. Transmission of *C. burnetii* by tick bite to animals has also been suggested [4].

In Ethiopia, Philip et al. [5] reported the first evidence of the existence of *C. burnetii* in ticks found on cattle. The seroprevalence of *C. burnetii* among slaughterhouse workers in an abattoir in Addis Ababa was reported to be 6.5% by complement fixation test [6]. According to recently published data, seroprevalence rates of *C. burnetii* determined by enzyme-linked immunosorbent assay (ELISA) were 31.6% in cattle, 90.0% in camels and 54.2% in goats. These values are higher than values reported in other parts of Africa [7].

Multispacer sequence typing (MST) [8] and multi-locus variable-number tandem repeat analysis (MLVA) [9], [10] are two PCR-based *C. burnetii* genotyping methods with high discriminatory power. These techniques do not require previous cultivation of *C. burnetii* under biosafety level 3 conditions and can be implemented directly on clinical and environmental samples e.g. for routine diagnostic purpose or during epidemic investigations.

The aim of the present study was to genetically characterize *C. burnetii* DNA samples originating from Ethiopian ticks and compare them to previously identified genotypes in order to obtain genotypic information from this poorly studied region.

## Materials and Methods

**Figure 1:**
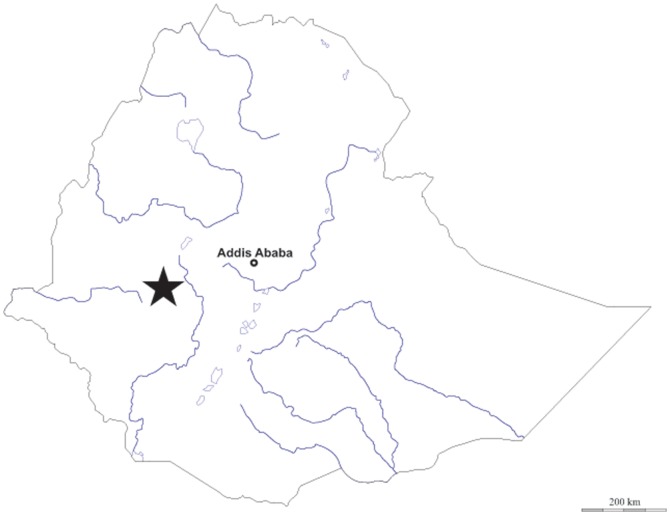
Map of Ethiopia showing the geographical origin of the samples (black star). (The blank map was downloaded from an open source [29].)

A total of 296 ticks (*Amblyomma variegatum*, n = 118; *A. cohaerens*, n = 100; *A. lepidum*, n = 2; *Rhipicephalus decoloratus*, n = 50; *R. evertsi*, n = 17; *R. praetextatus*, n = 8 and *Hyalomma rufipes*, n = 1) were collected from 109 cattle belonging to 18 different herds (zebu breed) in Didessa valley, south-western Ethiopia (09°05′N, 36°33′E - 7°40′N, 36°50E) in 2012 (Figure 1) [11]. Because sampling was part of the regular veterinary care and the field studies did not involve endangered or protected species, no specific permissions were required for these activities. Ticks were identified on the basis of their morphology by microscopy [12], and the DNA of each tick was extracted using the QIAamp DNA Mini Kit (Qiagen GmbH, Hilden, Germany) according to the tissue protocol in the manufacturers' instructions. In order to detect *C. burnetii* DNA and to estimate the amount of *C. burnetii* DNA (i.e. determine the cycle threshold (Ct) values), all tick samples were analyzed by a TaqMan based real-time PCR assay targeting the multi-copy IS*1111* insertion element of *C. burnetii* genome [13]. From the 32 positive samples, 8 cases (*Amblyomma cohaerens*, n = 6; *A. variegatum*, n = 2) which harbored sufficient amount of *C. burnetii* DNA (Ct<35) were selected for genotyping [11].

For the MST analysis ten selected spacer regions of the *C. burnetii* genome were amplified and sequenced according to the description of Glazunova et al [8]. The obtained sequences were trimmed, concatenated and aligned with genotypes from the MST database and previous publications using BioEdit Sequence Alignment Editor 7.1.11 [8], [14]–[16]. A Neighbor-joining phylogenetic tree was constructed using the maximum composite likelihood model and 1000 bootstraps with the Mega 5.05 software [17]. Sequence types (STs) were determined using data from the MST database and previous publications [8], [14], [15], [18], [19]. Unfortunately, the allele codes or nucleotide sequences of STs 35–51 are not publicly available therefore we were not able to include them in the phylogenetic analysis [18], [19].

MLVA was performed by single PCRs targeting six variable microsatellite markers. Ms27, Ms28 and Ms34 containing repeat units of six base pairs and Ms23, Ms24 and Ms33 containing repeat units of seven base pairs. The 3′-end-labelled forward and reverse primer sequences, and PCR conditions were applied as described before [20], [21]. The amplification products were run on an ABI 3100 Genetic Analyser and electropherograms were evaluated with the Peak Scanner Software 2.0 (Applied Biosystems Inc., Foster City, CA). DNA of the Nine Mile strain (RSA 493, Coxevac, Ceva Inc., Budapest, Hungary) was used as a reference. The repeat numbers of each marker were determined by extrapolation using the obtained length of the sample fragments relative to the obtained fragment-length of the reference strain. The updated coding convention was used for Ms33: nine imperfect repeat units were counted in the Nine Mile strain [22]. Novel MLVA types were determined if the described allele combinations had not been previously published in the *Coxiella* MLVA database nor described in any publication applying the same MLVA method [21]–[25]. Novel MLVA types were denominated in accordance with the nomenclature of the *Coxiella* MLVA database and previous publications [21]–[25].

## Results

**Figure 2:**
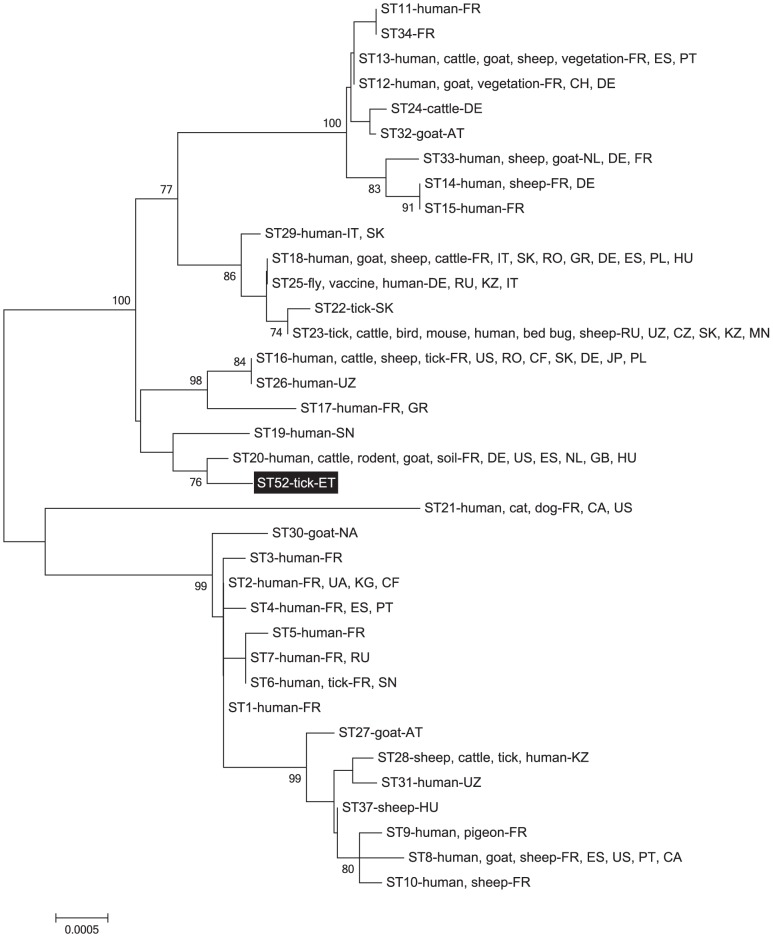
Neighbor-joining tree showing the placement and phylogenetic relationships of the novel sequence type (proposed ST 52) (highlighted area) from this study with known STs [8], [14], [15]. Bootstrap values of >70 are shown (1000 replicates). The scale bar represents the average number of substitutions per site. Isolate origins and sources are given according to previous publications [8], [13], [14], [18], [23]–[27], [30]–[32] using the following location codes: Austria (AT), Canada (CA), Central African Republic (CF), Czech Republic (CZ), Ethiopia (ET), France (FR), French Guiana (GF), Germany (DE), Greece (GR), Hungary (HU), Italy (IT), Japan (JP), Kazakhstan (KZ), Kyrgyzstan (KG), Mongolia (MN), Namibia (NA), Netherlands (NL), Poland (PL), Portugal (PT), Romania (RO), Russian Federation (RU), Senegal (SN), Slovakia (SK), Spain (ES), Switzerland (CH), Ukraine (UA), United Kingdom (GB), United States (US) and Uzbekistan (UZ).

The MST analysis identified one novel ST (proposed ST52) among the examined samples (Figure 2, Table 1). The allele codes of the newly identified ST were 3-9-6-1-5-4-4-10-6-5 for spacers Cox2-Cox5-Cox18-Cox20-Cox22-Cox37-Cox51-Cox56-Cox57-Cox61. This was not only a new combination of already known alleles of intergenic spacers, but a novel allele was also discovered, for the intergenic spacer Cox5, the proposed Cox5.9 (GenBank Accession number: KJ473948, KJ473949).

**Table 1:** MST and MLVA genotypes and information of *C. burnetii* strains detected in tick samples collected in Ethiopia.

ID number	Origin	Year	Tick species	Ct	Ms23	Ms24	Ms27	Ms28	Ms33	Ms34	MLVA type	MST type
**HSE-71**	Ethiopia, Didessa valley	2012	*Amblyomma variegatum*	26.6	6	12	2	7	10	10	24/AH	ST52
**HSE-73**	Ethiopia, Didessa valley	2012	*Amblyomma variegatum*	26.0	6	12	2	7	10	10	24/AH	ST52
**HSE-166**	Ethiopia, Didessa valley	2012	*Amblyomma cohaerens*	26.3	6	12	2	7	10	10	24/AH	ST52
**HSE-167**	Ethiopia, Didessa valley	2012	*Amblyomma cohaerens*	32.9	2	ND	ND	7	8	ND	partial	ND
**HSE-169**	Ethiopia, Didessa valley	2012	*Amblyomma cohaerens*	32.3	2	12	2	7	8	10	26/AI	ST52
**HSE-170**	Ethiopia, Didessa valley	2012	*Amblyomma cohaerens*	32.8	2	ND	ND	7	8	10	partial	ND
**HSE-209**	Ethiopia, Didessa valley	2012	*Amblyomma cohaerens*	30.0	2	12	2	7	8	10	26/AI	ST52
**HSE-210**	Ethiopia, Didessa valley	2012	*Amblyomma cohaerens*	32.4	ND	12	2	7	ND	10	partial	ND
**RSA493** *	USA	1935	*Dermacentor andersoni*		9	27	4	6	9	5		ST16

ND: not determined allele, probably because of insufficient DNA content,

*: Genotype of RSA493 strain (Nine Mile, GenBank accession number: AE016828) was determined by *in silico* analysis.

Two novel genotypes were identified with the MLVA method: type 24 (nomenclature of *Coxiella* MLVA database) or AH (nomenclature of previous publications) from both *Amblyomma* species and type 26 or AI only from *A. cohaerens*. The allele code for markers Ms23-Ms24-Ms27-Ms28-Ms33-Ms34 in genotype 24/AH was 6-12-2-7-10-10 while in genotype 26/AI it was 2-12-2-7-8-10 [22].

In the case of three *A. cohaerens* samples only partial MLVA genotypes were obtained, probably because of the insufficient DNA load (high Ct values). The combinations of the identified alleles in samples HSE-167 and HSE-170 were identical to the complete MLVA genotypes 26/AI, suggesting that they belong to the same genotype, while sample HSE-210 could belong to either of the newly determined MLVA types (26/AI or 24/AH).

## Discussion

Both typing methods revealed novel *C. burnetii* genotypes when compared to genotypes with publicly available genetic data [8], [14], [15], [21]–[25]. However, since several MST genotypes (STs 35–51) were determined and denominated by previous publications without presenting the allele codes or nucleotide sequences [18], [19], we cannot rule out the possibility that the novel ST documented in the present study may eventually be identical with one of them. The lack of data harmonization between the MST and different MLVA systems presents a further challenge.

Information on the genotypic characteristics of *C. burnetii* isolates originating from Africa is restricted. Up to now ST2 and ST16 from Central Africa, ST30 from Namibia and ST6, ST19 and two partial STs (ST 35 and 36) from Senegal are the only described *C. burnetii* genotypes from Africa, identified in ticks and human samples [8], [19], [26]. The newly discovered ST52 is closely related to the widely distributed ST20 (Figure 2). ST20 has been reported from Germany, France, Hungary, the Netherlands, Spain, the United Kingdom and the United States. This genotype is most often associated with cattle samples, but it occasionally infects other species as well [8], [15], [25], [27]. ST52 is also related to ST19, described earlier from human clinical samples from Senegal [8].

ST52 was further discriminated into two newly identified MLVA types (24/AH and 26/AI). The comparison of the two novel genotypes with others described elsewhere revealed high similarity with genotypes originating from cattle. For instance the novel type 24/AH and type 25/L previously identified from Slovakian cow milk differs only in one out of the six loci (12 versus 13 copies at locus Ms24) [23].

Both typing methods revealed that these novel *C. burnetii* genotypes are closely related to genotypes previously described from cow samples from other parts of the globe. This finding is congruent with the source hosts of the Ethiopian ticks analyzed in the present study, as these were also collected from cattle. Therefore, our results provide additional evidence of the presumed host-specific evolutionary adaptation of *C. burnetii* [15], [28].

In conclusion, the present study provides genetic information about *C. burnetii* from the poorly studied African continent. One novel MST type (proposed ST52) and two novel MLVA types (24/AH and 26/AI) have been identified among the eight examined *C. burnetii* DNA samples of Ethiopian ticks collected from cattle. Our results are bringing to light some missing parts of *C. burnetii* phylogeny and they also provide further evidence for the presumed host-specific adaptation of this agent.
